# A Case Report of Advanced Cervical Cancer in a Patient Non-compliant With Age-Appropriate Screening

**DOI:** 10.7759/cureus.21744

**Published:** 2022-01-30

**Authors:** Shobha Mandal, Sohaib Shabih, Jagdesh Kumar, Surendra Shah

**Affiliations:** 1 Internal Medicine, Guthrie Robert Packer Hospital, Sayre, USA; 2 Hematology and Oncology, Guthrie Robert Packer Hospital, Sayre, USA

**Keywords:** screening, pap smear, cannonball mass, lung metastasis, cervical carcinoma

## Abstract

Cervical carcinoma is one of the preventable malignancies in the United States. Age-appropriate screening has decreased the incidence of cervical cancer. A multitude of age-appropriate screening methods is available including Papanicolaou (Pap) smear cytology, human papillomavirus (HPV) DNA testing, and visual inspection tests. Patients who are not up to date with the screening can remain asymptomatic until the advanced stage like in the case of our patient. We present a 59-year-old female, who came in with progressively worsening shortness of breath on exertion, chest tightness, significant weight loss, and vaginal bleeding for the past six months. On investigations, she was found to have cannonball metastases in the lung. The patient remained critically ill during her course of hospital stay and eventually passed away.

## Introduction

Cervical cancer is one of the major causes of cancer-related death in women worldwide [1]. It is the fourth most prevalent cause of malignancy in women after breast, colorectal, and lung cancer [2]. The incidence of cervical cancer and mortality rate has declined by 70% in the United States since the 1950s because of age-appropriate screening [3]. Among the several risk factors, human papillomavirus (HPV) types 16, 18, 31, 33, and 45 are the leading cause of cervical neoplasia [4]. Other risk factors are smoking, low socioeconomic status, early age at the first coitus, multiple sexual partners, and multiparity [5]. 

## Case presentation

A 59-year-old female with no significant past medical history, non-compliant with any age-appropriate cancer screening, presented to the Emergency Department with progressively worsening shortness of breath on exertion with productive cough and chest tightness for the past six weeks. She also endorsed malaise, progressive fatigue, anorexia, chronic pelvic discomfort, and weight loss of 70 lbs in the last year. She denied any vaginal bleeding or discharge. She had not seen a physician in the last eight years and never had a Papanicolaou (Pap) smear, mammogram, or colonoscopy done.

**Figure 1:**
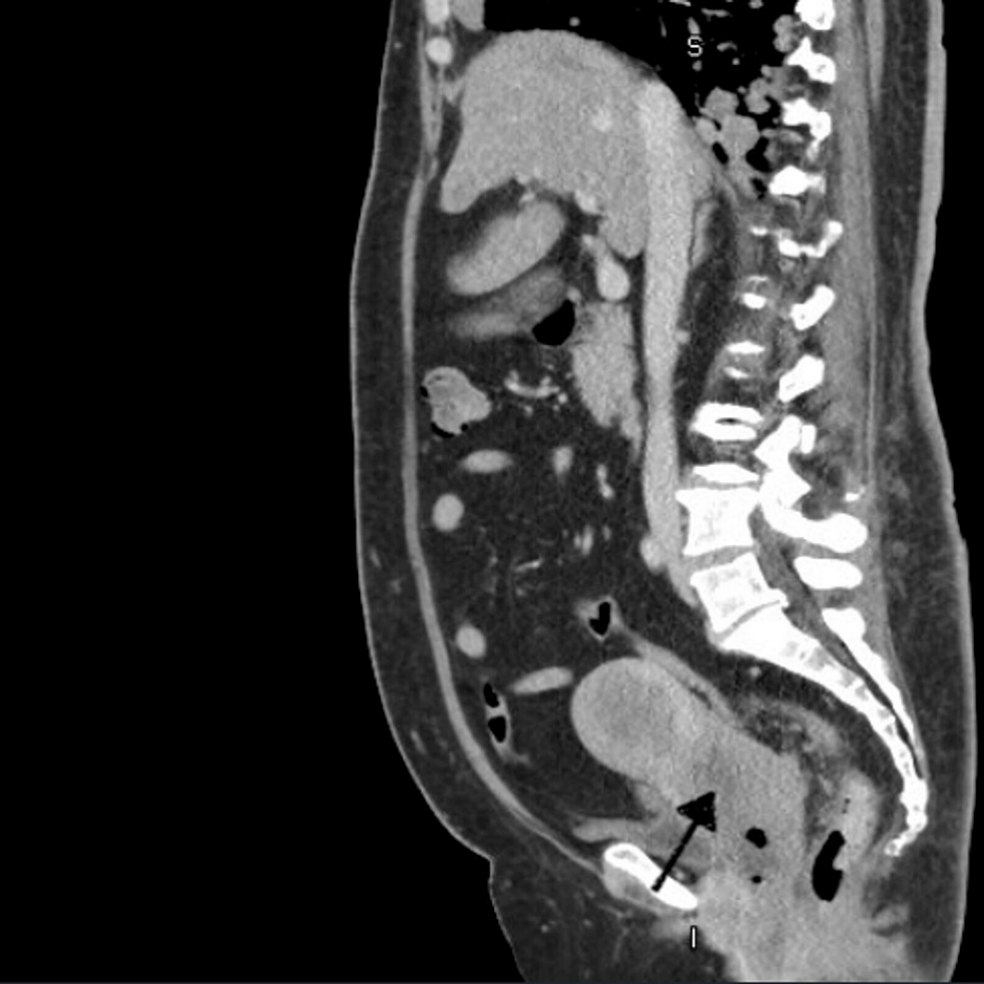
CT abdomen pelvis (sagittal view) showing soft tissue mass involving the cervix with nonhomogeneous density suggesting an underlying cervical mass (black arrow)

On arrival, the patient was in respiratory distress, requiring 6-8 L oxygen through nasal canula but otherwise, hemodynamically stable. On systemic examination, she appeared cachectic, with bilateral coarse breath sounds on lung exam and right lower quadrant tenderness on abdominal examination. Blood work showed leukocytosis (WBC count: 20K cell/Liter), lactic acidosis (4.6 mmol/L), hypercalcemia (serum total calcium 12.6 mg/dl), elevated d-dimer (1.6 mcg/mL). Urinalysis was significant for hematuria but negative for pyuria. Serum lipase and amylase levels were unremarkable. Acid-base gas showed pH 7.43/PCO2 40/ PO2 59. Computed Tomography (CT) abdomen pelvis with intravenous contrast showed cervical soft tissue mass measuring up to 7x7 cm with an ill-defined feature, suggestive of neoplasm (Figure 1).

**Figure 2:**
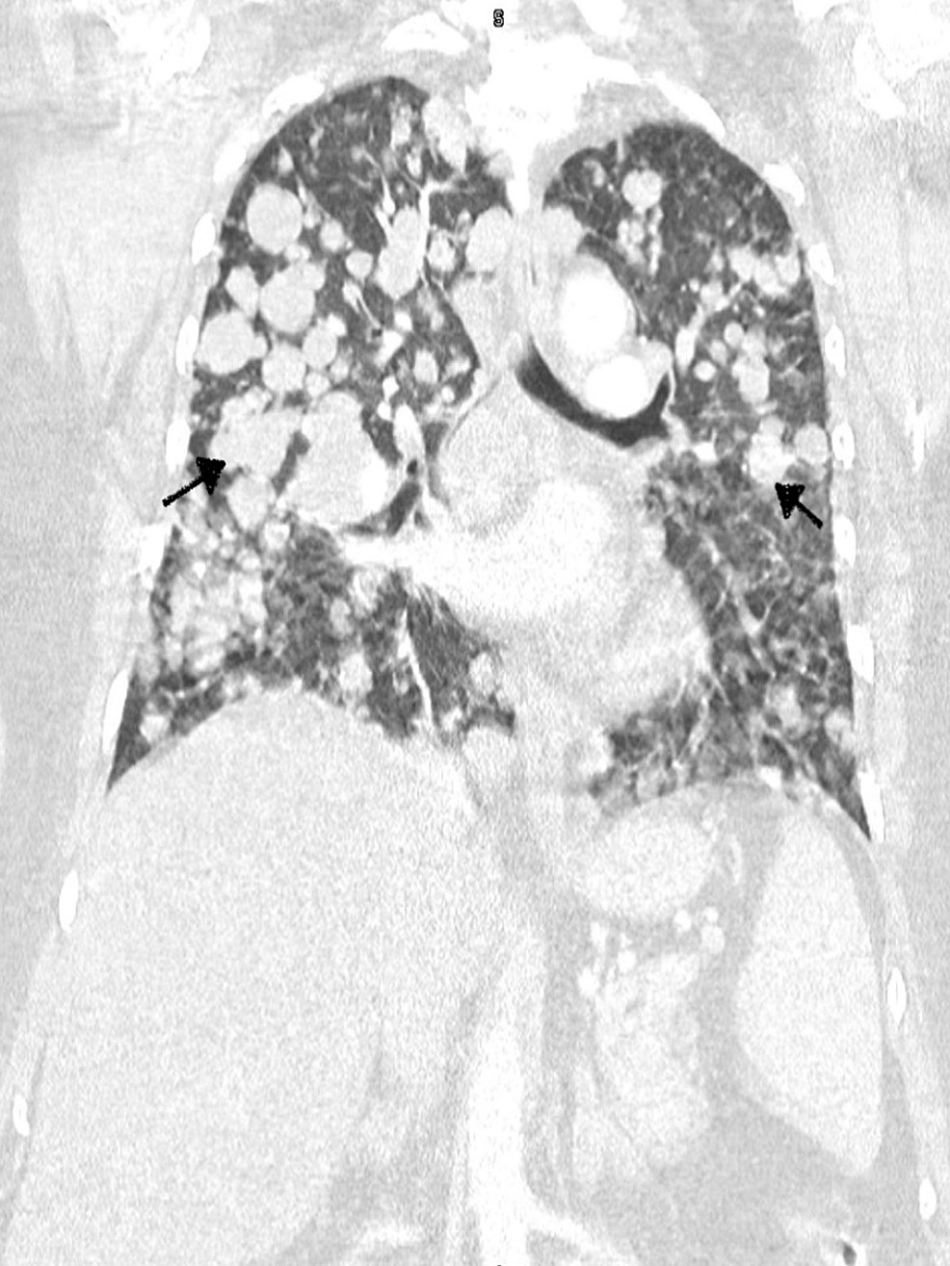
CT chest showing multiple lesions in the lung suggestive of metastasis (black arrows)

CT of the chest was obtained, which showed multifocal mediastinal masses measuring approximately 3 cm in the pre-tracheal and 35 x 37 mm in the SUV carinal region, bilateral hilar adenopathy, numerous nodular masses throughout the lung, and 50% predominance of focal lung consolidation (Figure 2). 

Coronavirus disease 2019 (COVID-19) test was done, which was negative. Infectious workup, including blood culture, sputum culture and gram stain, legionella, and pneumococcal antigen tests were obtained, and the patient was started on empiric broad-spectrum antibiotics. As there was concern about cervical cancer with metastasis to the lungs, she was seen by a gynecologist and was planned for a biopsy of the cervical mass. Hematology/oncology was on board as well and recommended a biopsy of the cervical mass with cytology, and HPV testing. If the biopsy comes positive for cervical cancer, she was planned to be started on a cisplatin-based chemotherapeutic regimen with the addition of checkpoint inhibitor therapy if the programmed death-ligand 1 (PD-L 1) biomarker was positive. 

During the hospital stay, the patient continued to have worsening hypoxemia, with alteration in her mental status, and eventually developed multiple organ failure with renal dysfunction, transaminitis, and lactic acidosis. She was placed on bilevel positive airway pressure (BiPAP), but her oxygen requirements continued to worsen. The patient was Do-Not-Intubate (DNI)/Do-Not-Resuscitate (DNR). Palliative medicine was on board for goals of care discussion due to the patient’s declining clinical condition. The family decided to proceed with comfort care measures and the patient passed away. We were not able to obtain the biopsy of the cervical mass as we wanted the patient condition to be more stable.

## Discussion

The cervix is the lower part of the uterine cavity and is covered with two types of cells: the glandular and the squamous cells. The junction of these two types of cells is known as the transformation zone. Most cervical cancer originates from the transformation zone. Squamous cell carcinoma (95%) and adenocarcinoma (5%) are the two major histological types of an epithelial tumor of the cervix, but in rare cases, the tumor can also be of a non-squamous variant including adenosquamous carcinoma, neuroendocrine carcinoma, glassy cell carcinoma [6]. 

Metastasis in cervical cancer occurs by hematogenous and lymphatic spread. Lung, bone, liver, and brain are the most common organs of cervical cancer metastasis [7]. The lung is the most common metastasis site of the cervix and metastasis to the lung occurs because of the hematogenous spread [8]. The incidence of lung involvement increases stage-wise; 3.2% in stage I, 5.0% in stage II, 9.4% in stage III, and 20.9% in stage IV disease [9]. In the Surveillance, Epidemiology, and End Results (SEER) database study from 2020, it was found that patients with age greater than 65, non-squamous histology type cervical cancer, pelvic lymph nodes metastases, poor differentiation, other organ metastasis were at higher risk for lung metastasis [ 10]. 

Patients with early-stage cervical cancer are mostly asymptomatic but few of those who are symptomatic, present with lower abdominal pain, postcoital vaginal bleeding, abnormal menstrual bleeding, post-menopausal bleeding, offensive vaginal discharge, and dyspareunia [11]. Vaginal discharge may be watery, mucoid, or purulent. Patients in the advanced stage of cancer present with symptoms of urinary symptoms, lower abdominal pressure, hematuria, and hematochezia depending on the site of metastasis. 

Screening strategies for cervical cancer include Pap smear testing alone, primary HPV testing alone, or co-testing (with Pap and HPV testing). For patients under 21, screening is not required regardless of the age of initiation of sexual activity. In patients between 21-29, screening is initiated at age 21 with cervical cytology every three years. For patients aged 30 to 65, either Pap testing alone every three years or co-testing (PAP and HPV testing combined) every five years is recommended. For patients who are above 65, the decision to continue screening depends on whether the patient has had an adequate prior screening, life expectancy, and preferences in a shared decision-making discussion. Symptomatic patients should have Pap smear testing as part of a diagnostic workup, regardless of prior screening results [12]. 

It has been noted that more than half of patients who develop cervical cancer have not been screened adequately. In most of the patients with invasive cervical cancer, there was no Pap smear obtained in the past five years [13]. Strategies that can be used to increase screening rates include actively inviting patients to schedule timely appointments for cervical cancer screening. Urgent care clinical visits can be used as an opportunity to screen patients who are unlikely to otherwise comply with cervical cancer screening recommendations [14,15].

Cervical cancer can be diagnosed by colposcopy, fluorescence spectroscopy, molecular diagnostic methods like HPV DNA test, nuclear aneuploidy detection. Further testing with pelvic MRI and positron emission tomography-computed tomography (PET-CT) should be performed for locally advanced diseases. In patients with advanced cervical carcinoma, chest x-ray and brain CT can be done to rule out metastasis to the lung or brain [16]. Early-stage cervical cancer has a good prognosis because of the advancement in the treatment including surgery, chemotherapy, or radiotherapy, but advanced cancer with metastasis has a poor prognosis with a median survival of 8-13 months [17]. 

## Conclusions

In conclusion, most patients with advanced cervical cancer with lung metastasis are asymptomatic and are found to have incidental mass on routine chest x-rays or CT. As patients with advanced cancer have a poor prognosis, age-appropriate screening, early identification of patients at high risk for lung metastases, and early intervention can be lifesaving. 
